# Outcome of a cohort of severe cerebral venous thrombosis in intensive care

**DOI:** 10.1186/s13613-016-0135-7

**Published:** 2016-04-12

**Authors:** Benjamin Soyer, Marco Rusca, Anne-Claire Lukaszewicz, Isabelle Crassard, Jean-Pierre Guichard, Damien Bresson, Joaquim Mateo, Didier Payen

**Affiliations:** 1 Department of Anesthesiology and Critical Care and SMUR, Hôpital Lariboisière, AP-HP, 2 Rue Ambroise Paré, 75010 Paris, France; 2 Université Diderot Sorbonne Paris Cité, Paris, France; 3 Département Hospitalo-Universitaire (DHU) Neuro-Vasculaire, P R E S Paris Sorbonne Cité, Paris, France; 4 Service of Neurology, Hôpital Lariboisière, AP-HP, Université Paris 7 Denis Diderot, Paris, France; 5 Department of Neurovascular Imaging, Hôpital Lariboisière, AP-HP, Université Paris 7 Denis Diderot, Paris, France; 6 Service of Neurosurgery, Hôpital Lariboisière, AP-HP, Université Paris 7 Denis Diderot, Paris, France

**Keywords:** Severe cerebral venous thrombosis, Intracranial hypertension, Intracranial hematoma, Decompressive craniectomy, Endovascular therapies, Neuro-resuscitation, Multimodal monitoring

## Abstract

**Background:**

Severity of cerebral venous thrombosis (CVT) may require the transfer to intensive care unit (ICU). This report described the context for CVT transfer to ICU, the strategy of care and the outcome after 1 year.

**Methods:**

Monocentric cohort of 41 consecutive CVT admitted in a French ICU tertiary hospital (National Referent Center for CVT). Data collected are as follows: demographic data, clinical course, incidence of craniectomy and/or endovascular procedures and outcome in ICU, after 3 and 12 months.

**Results:**

47 years old (IQ 26–53), with 73.2 % were female, having a SAPS II 41 (32–45), GCS 7 (5–8), and at least one episode of mydriasis in 48.8 %. Thrombosis location was 80.5 % in lateral sinus and 53.7 % in superior sagittal sinus; intracranial hematoma was present in 78.0 %, signs of intracranial hypertension in 60.9 %, cerebral edema in 58.5 % and venous ischemia in 43.9 %. All patients received heparin therapy, and 9 cases had endovascular treatment (21.9 %); osmotherapy (53.7 %) and decompressive craniectomy (16 cases, 39 %) necessary to control intracranial hypertension. Ten patients/41 (24.4 %) died in ICU and 18/31 (58.1 %) were discharged from ICU with outcome 0–3 of mRS. After 12 months, 92 % of survivors (23/25) had a mRS between 0 and 3. The proportion of death was 31.7 % at 1 year.

**Conclusions:**

The large proportion of acceptable outcome in survivors, which continue to functionally improve after 1 year, motivates the hospitalization in ICU for severe CVT. For similar CVT severity, craniectomy did not improve outcome in comparison with the absence of craniectomy.

**Electronic supplementary material:**

The online version of this article (doi:10.1186/s13613-016-0135-7) contains supplementary material, which is available to authorized users.

## Background

Cerebral venous thrombosis (CVT) is a difficult diagnosis of a rare brain vascular disease accounting for 0.5–1 % of all strokes [1, 2]. In the largest international multicenter cohort of CVT including 624 patients (ISCVT) with large spectrum of presentation, Ferro et al. showed a mortality rate of 4.3 % at the acute phase and 8.3 % at the last follow-up (16 months) [3]. The authors mentioned 26 cases (4.1 %) with severe clinical presentations, but with elusive clinical information and care strategies and intensive care unit (ICU) hospitalization or not. Using the same database, the authors have reported [4] the main causes of death. Transtentorial herniation due to a unilateral focal mass effect or to diffuse edema and multiple parenchymal lesions were independent predictors of death. Coma [5], mental disturbance of deep CVT thrombosis, right intracerebral hemorrhage and posterior fossa lesion [3] were also associated with poor outcome. Recently, a study from multicenter registry had reported the outcome of 69 patients having decompressive craniectomy [6]. If 31 patients came from the previously published registry, 34 were new cases or case reports. Although no information about incidence of ICU hospitalization, neuro-resuscitation and neuro-monitoring was mentioned, authors have reported unfavorable outcome (50 % of modified Rankin score (mRS) 4 or 5; or 42 % to die) when bilateral lesions were present compared to 11 % in unilateral lesions.

In our knowledge, there is no article on strategy of care and outcome of CVT patients requiring ICU admission. This study reports the profile of severe CVT transferred to ICU, the monitoring used, the strategy of care (supportive and instrumental interventions) with the observed complications and outcome in ICU and after 1 year.

## Methods

### Patients

Among the patients admitted to the neurological department of our institution (National Reference Center for CVT), some cases were transferred from 2002 to 2015 to our ICU for the following indications: severe alteration of consciousness or coma, status epilepticus, intracranial hypertension and/or requirement of mechanical ventilation. The diagnosis of CVT was usually established before ICU admission in all cases by CT (computerized tomography), venography, MRI (magnetic resonance imaging) or MRI combined with MR venography (MRV) and/or angiography.

The recorded clinical data were age, sex, medical history with special focus on malignancy and other known risk factors [3, 7, 8]. Clinical severity at ICU admission was evaluated by SAPS II, coma was graded with Glasgow Coma Scale (GCS), and presence of seizure, mydriasis and elevated intracranial pressure (ICP) (CT scan) were collected, as well as routine laboratory tests. The delay between the onset of neurological symptoms and ICU admission or between the diagnosis and ICU admission was also recorded.

Brain imaging techniques were as follows: CT scan, MRI and MRV interpreted by an independent radiologist (JPG). This evaluation had to mention the number and topography of the venous and sinus thrombosis, presence of hematoma, the associated brain damages related to venous congestion and presence of hydrocephalus.

The ICU strategy of care aimed to improve cerebral perfusion and oxygenation by classic means (central temperature <38 °C; arterial partial pressure in CO_2_ PaCO_2_ = 40; cerebral perfusion pressure >65 mmHg; and rigorous control of fluid balance). Monitoring consisted in invasive intra-arterial pressure (radial or femoral artery), end-tidal CO_2_ and middle cerebral artery transcranial Doppler velocities (Waki, Atys Medical, France; Ultrasound Echo-Doppler Vivid, General Electrics, USA). Treatment of CVT was based on heparin anticoagulation at therapeutic dose for all patients. The resistance to heparin was carefully checked by the delay to reach an adequate aPTT (activated partial thrombin time) (between two to three times of control). Interventional therapy such as invasive endovascular and neurosurgical lifesaving techniques was discussed collegially. Since venous circulation has a low intravascular pressure, moderate intracranial hypertension may impair venous flow, justifying the craniectomy. Endovascular treatment was decided when anticoagulation and control of cerebral perfusion and oxygenation were adequate but with occurrence of signs of uncontrolled intracranial hypertension. A CT scan was always performed before intervention to analyze the mass effect or the disappearance of cortical sulci and herniation.

Neurological complications were quoted and corresponded to a new episode of seizure, a new focal sign, new episode of hemorrhage or worsening of hematoma and post-interventional intracranial infection. The following extra-neurological complications were collected: remote thromboembolic episode, anticoagulation complications (extracranial bleeding and heparin-induced thrombocytopenia) and nosocomial infections.

The outcome study had consisted in evaluation of ICU death, a mRS at ICU discharge, repeated at 3 and 12 months and more when possible. Post-ICU outcome data were obtained from external consults made by a neurologist. As previously reported [6], the functional outcome was classified as follows: good recovery: mRS from 0 to 3; poor recovery: mRS 4 or 5; and death (mRS 6). The impact of craniectomy and/or endovascular treatment on death rate and functional outcome was also evaluated.

### Statistical analysis

Clinical and biological quantitative variables were expressed as median and 25th–75th interquartiles. Intergroup comparisons were made by a Mann–Whitney test for quantitative variables and Chi-square test with Fischer correction for qualitative variables. A *p* < 0.05 was considered as statistically significant.

## Results

### Admission in ICU

**Fig. 1:**
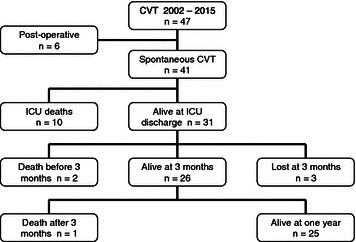
Flowchart of the CVT cohort along the survey period. Along the 13-year survey period, 47 patients were recruited. Six were excluded because a postoperative context (ENT surgery and one meningioma surgery). “Spontaneous medical” CVT were finally collected: 10 died in ICU, 3 died after ICU discharge and 3 were lost; 25 are currently alive and were studied for functional outcome after at least 1 year evolution

Forty-seven patients with severe CVT were admitted in the ICU from 2002 to 2015 (Fig. 1). Six patients were excluded from the analysis because CVT occurred in a surgical context of ear–nose–throat infection or facial cellulitis (*n* = 5) or complicating intracranial surgery (meningioma, *n* = 1). Characteristics of the 41 remaining patients are summarized in Table 1, with a mean age 47 years (26–53), 73.2 % female, a SAPS II 41 (32–45). The main risk factors for CVT were summarized in Additional file 1. Reasons for ICU admission were a rapid alteration in consciousness [GCS 7 (5–8)], associated with seizure for 20 patients (48.8 %) and mydriasis for 20 patients (48.8 %). Twenty-five patients (60.9 %) had marked CT scan symptoms of elevated ICP. Before ICU admission, around two-thirds of patients received heparin treatment. Early intubation (within 48 h) was necessary in 37 patients (90.2 %).

**Table 1:** ICU admission main characteristics, supportive therapies, outcome for all patients and ICU survivors or dead

	Total CVT *n* = 41	ICU deaths *n* = 10	ICU survivors *n* = 31	*p* value
Age (years)	47 (26–53)	44.5 (30.8–52)	47 (24–51.5)	0.82
Sex female (%)	30 (73.2)	10 (100)	20 (75.6)	0.039
SAPS II	41 (32–45)	42.5 (41–52.5)	35 (31.5–44.5)	0.072
Delay for diagnosis (days)	3 (1–7)	3 (1–5.5)	3 (1–7)	0.73
Delay diagnosis–ICU admission (days)	1 (0–3)	0 (0–1)	1 (1–4.5)	0.029
GCS (lowest)	7 (5–8)	4 (3–6)	8 (6–9)	0.0004
Seizure in ICU [*n* (%)]	18 (43.9)	4 (40)	14 (45.2)	1.00
Mydriasis [*n* (%)]	20 (48.8)	10 (100)	10 (32.3)	0.0001
Hematoma [*n* (%)]	32 (78.0)	10 (100)	22 (70.9)	0.083
Heparin before admission [*n* (%)]	25 (60.9)	5 (50)	20 (75.6)	0.47
aPTT ratio > 2 within 48 h [*n* (%)]	30 (80.5)	5 (50)	25 (80.6)	0.098
Craniectomy (n (%))	16 (39.0)	5 (50)	11 (35.5)	0.47
Endovascular treatment [*n* (%)]	9 (21.9)	3 (30)	6 (19.4)	0.66
CSF shunt [*n* (%)]	7 (17.7)	2 (20)	5 (16.1)	1.00
IV norepinephrine [*n* (%)]	26 (63.4)	9 (90)	17 (54.8)	0.063
MAP min day 1 (mmHg)	76 (66–86)	83 (70–98)	76 (65–83)	0.23
MAP min day 2 (mmHg)	75 (71–84)	73 (59–91)	76 (72–83)	0.56
Mechanical ventilation [*n* (%)]	37 (90.2)	10 (100)	27 (87.1)	0.55
Fluid balance day 1 (mL)	−188 (−482 to +393)	−350 (−1224 to −145)	−188 (−440 to +429)	0.45
Fluid balance day 2 (mL)	247 (−756 to +809)	−50 (−679 to +878)	344 (−782 to +797)	0.59
Natremia day 1 (mM)	138 (135–142)	139 (137–141)	137 (135–142)	0.64
Platelet count day 1 (10^9^/L)	231 (181–305)	210 (162–239)	240 (205–309)	0.23
Glycemia day 1 (mM)	7.1 (6.1–8.3)	7.5 (6.2–10.8)	7.1 (5.9–7.8)	0.19
mRS at discharge	4 (3–5)	6	3 (2.5–4)	–
mRS at 3 months (*n* = 23)	3 (2–4)	–	3 (2–4)	–
mRS at 12 months (*n* = 18)	2 (1–3)	–	2 (1–3)	–
mRS at last follow-up	2 (1–6)	6	2 (0.5–3)	–

Median and interquartile or proportion *p* < 0.05 were considered as statistically significant. *CVT* cerebral vein thrombosis, *ICU* intensive care unit, *SAPS II*: Simplified Acute Physiology Score II, *GCS* Glasgow Coma Scale, *aPPT* activated partial thromboplastin time, *CSF* cerebral spinal fluid. *IV* intravenous, *MAP* mean arterial pressure, *Min* minimal, *mRS* modified Rankin score

### Topography of brain lesions

The diagnosis of CVT was made before ICU admission by imaging techniques (CT scan and angio-CT scan, MRI and MRV) in 100 %. A combination of imaging techniques to diagnose CVT was necessary in 73.2 % of patients. Topography for thrombosis was listed in Additional file 2: 80.5 % being in lateral sinuses, 53.7 % in the superior sagittal sinus and 26.8 % in a deep cerebral vein, a recognized risk factor for death. Thrombosis in 3 or more venous axes was observed in 51.2 %. The associated lesions are listed in Table 2, with hematoma in 78.0 %, cerebral edema in 58.5 % and venous ischemia in 43.9 %. Unilateral lesions (hematoma and parenchymal ischemia) were present in 43.4 %, and bilateral lesions were present in 36.6 %.

**Table 2:** Types of brain damage related to CVT in whole cohort, and ICU survivors or death

Brain damages	All CVTs *n* = 41 (%)	ICU deaths *n* = 10 (%)	ICU survivors *n* = 31 (%)	*p* value
Hematomas and hemorrhagic transformations	32 (78.0)	10 (100)	22 (70.9)	0.083
Left hemisphere	10 (24.4)	3 (30)	7 (22.6)	0.68
Right hemisphere	6 (14.6)	1 (10)	5 (16.1)	1.00
Both hemispheres	14 (34.1)	5 (50)	9 (29.0)	0.26
Median structures	9 (21.9)	3 (30)	6 (19.4)	0.66
Posterior fossa	3 (7.3)	1 (10)	2 (6.5)	1.00
≥2 hematomas	16 (39.0)	6 (60)	10 (32.3)	0.15
Intracranial hypertension	25 (60.9)	10 (100)	15 (48.4)	0.003
Cerebral edema	24 (58.5)	7 (70)	17 (54.8)	0.48
Venous ischemia	18 (43.9)	6 (60)	12 (38.7)	0.29
Subarachnoid hemorrhage	8 (19.5)	3 (30)	5 (16.1)	0.38
Hydrocephaly	5 (12.2)	1 (10)	4 (12.9)	1.00

*p* < 0.05 was considered as statistically significant

### ICU treatment

All patients were treated in ICU with unfractioned heparin targeting an aPTT ratio >2. This goal was obtained within the first 48 h in 30 patients (80.5 %). Cerebral perfusion was evaluated by transcranial Doppler in 32 patients (78.0 %) helping to optimize the strategy of care. In few cases (*n* = 8), intracranial pressure was monitored despite the risk of bleeding. When decided, the improvement in cerebral perfusion pressure required norepinephrine infusion in 26 patients (63.4 %), and 22 patients (53.7 %) had an osmotherapy to reduce cerebral edema.

### Interventional treatment for thrombosis

Sixteen patients (39 %) had an early (48 h) craniectomy that was associated with evacuation of hematoma (*n* = 5) and cerebral spinal fluid (CSF) drainage (*n* = 4). Two patients had the 3 interventions. Nine patients underwent in situ endovascular treatment because of neurological deterioration or lack of improvement: 3 had a thrombectomy alone; 1 had a thrombolysis only; and 5 had a combination of the 2 treatments. In 6 patients, the venous circulation was restored with only 1 major parenchymal bleeding. The presence of severe intracranial hypertension is required to evacuate hematoma (*n* = 2), to perform a CSF drainage (*n* = 7) and/or to perform a craniectomy [uncontrolled pressure (*n* = 4)].

### Outcome

The total of dead patients after 1 year was 13/41 (31.7 %). Ten patients died in ICU (24.4 %) having similar delay between initial symptoms and diagnosis compared to survivors, with a more rapid deterioration to be transferred to ICU (*p* = 0.029) (Table 1). The clinical profile of dead patients compared to survivors was: predominance of female (*p* = 0.039), a worse initial GCS [4 (3–6); *p* = 0.0004], a more frequent mydriasis (100 vs. 32.3 %, *p* = 0.0001) with more frequent signs of intracranial hypertension on imaging techniques (*p* = 0.003), with a trend for more frequent use of norepinephrine (90 and 54.8 %, respectively) and more difficulty achieving therapeutic anticoagulation (50 vs. 80.6 %, respectively). Location of thrombosis, topography of parenchymal lesions or therapeutic strategy did not differ between the two groups (Tables 1, 2, and Additional file 2), with a trend for more frequent intracranial hematomas (100 and 70.9 %, respectively). Death rate was surprisingly the same when lesions were unilateral or bilateral (12.2 vs. 12. %). The 16 patients who underwent decompressive craniectomy did not differ from others CVT at ICU admission, but received norepinephrine more frequently (*p* = 0.018). They had frequent hematomas (93.8 and 68 %, respectively) and an anticoagulation more difficult to be adequate within 2 days (56.3 and 84 %, respectively). These patients had a similar 1-year outcome compared to patients who were not treated by craniectomy, although the mRS at ICU discharge and after 3 months tended to be worse (Additional file 3). The 9 patients who underwent an endovascular treatment were similar to the others, but tended to have more mydriasis (77.8 vs. 40.6 %, *p* = 0.067) and received more frequently norepinephrine infusion (*p* = 0.015). Their mortality rate in ICU and the mRS at discharge did not differ from the other patients (Additional file 3).

Despite maximal treatment, among the 10 patients who died in ICU, 9 had neurological complications such as uncontrolled ICHT or brain herniation and 1 patient died from massive pulmonary embolism. Observed ICU complications were as follows: seizures (*n* = 18); deterioration of hemorrhagic lesions under heparin treatment (*n* = 8, 19.5 %); nosocomial infections (craniectomy wound infection or ventriculitis, *n* = 4); pneumonia (*n* = 18, 43.9 %); urinary tract infections (*n* = 5, 12.2 %); and complications related to heparin treatment (*n* = 5): heparin-induced thrombocytopenia (*n* = 1), gastric hemorrhage (*n* = 1), gynecologic bleeding (*n* = 1), mesenteric hematoma with hemorrhagic shock (*n* = 1) and inguinal hematoma (*n* = 1).

**Fig. 2:**
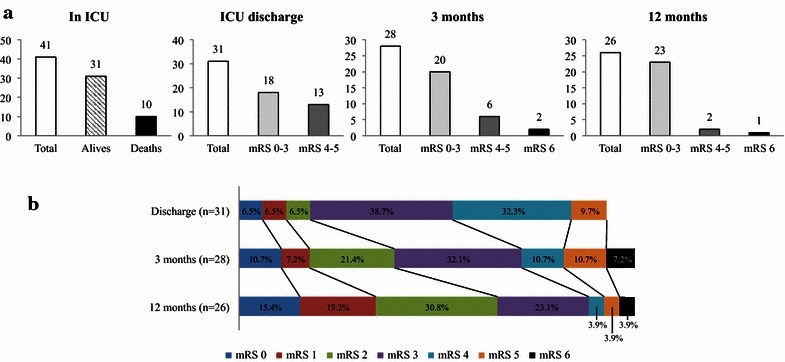
Outcome of severe CVT from ICU discharge to the next 12 months. **a** The evolution of the distribution of patients along the survey period with white bars for the number of the patients. At each period of monitoring (in ICU, at ICU discharge, 3 and 12 months), patients are divided into: group “good recovery” mRS 0–3 (*light gray bars*), “poor recovery” mRS 4–5 (*dark gray bars*) and death mRS 6 (*black bars*). **b** The distribution of mRS along the survey period at ICU discharge, 3 and 12 months. Proportion for functional ranking was calculated as a ratio between mRS value divided by the number of patients at the time of evaluation. Patients lost after discharge were excluded

After ICU discharge (3, 12 months, or later), 3 additional deaths occurred for different reasons such as brain tumor, limitation of treatment or unknown context. Among the 31 patients discharged from ICU, 18 patients (58.2 %) were classified as “good outcome” (mRS 0–3). Since 3 patients were lost for the follow-up at 3 months, the proportions were adjusted accordingly. mRS further improved along the survey period to 74.1 % (*n* = 20/23) at 3 months and 88.5 % (*n* = 23/26) at 1 year. Only 2 patients only had a mRS at 4–5 after 1 year (Fig. 2). For the patients who could be followed over 1 year (*n* = 19), 57.9 % (11/19) continued to improve the median mRS from 2 to 1 (median survey: 28 months).

## Discussion

The study first reports a cohort of patients with severe CVT admitted and treated in ICU, which provides information on clinical profile, management and outcome. The mortality of our cohort is higher than the one reported on patients having craniectomy [6], for whom little is known on clinical situation. However, the survivors of our cohort of very severe patients had a functional outcome almost identical to the one published in the ISCVT cohort having only 4 % of severe presentation [3]. Our patients had a mRS between 0 and 3 in 88.5 %, a proportion similar to the 89.5 % in ISCVT cohort, who were not all treated in ICU. This suggests an effectiveness of both ICU care with tight monitoring associated with multi-disciplinary strategy discussion.

### Characteristics and outcome

Apart from the severity, our cohort appeared similar for clinical characteristics to previous publications grouping all types of CVT. The patients were predominantly female, young, with multi-site thrombosis (thrombosis of superior sagittal sinus in 53.7 and 80.5 % in lateral sinuses) and classic risk factors for CVT, but with different proportions compared to previous reports [3]. In the ISCVT cohort, 31 % had a lesion dominated by hematoma, which was 78.0 % in our cohort, an incidence that may explain the severity justifying ICU hospitalization. Few patients in ISCVT study had criteria for ICU admission (1.1 % of mechanical ventilation, 1.4 % surgery and 1.6 % shunts), proportions that largely differed from our cohort. Among the 32 patients having hematoma in our cohort, 10 patients died (31.3 %) during ICU stay. All patients deceased in ICU had an intracranial hematoma associated with lateral sinus thrombosis, previously reported to be a risk of death [4, 9]. Importantly, the unilateral or bilateral lesions had non-difference in mortality (12.2 % for both), a very different results when compared to the study on multiple registry [6]. Although being more severe in our cohort, the survivors had similar rate of good outcome around 90 % after at least 1 year of evolution than those reported by others [3, 10–12].

Despite a worse functional outcome at ICU discharge, survivors had a positive evolution along the survey period: at the 1 year, only two patients over 26 (7.8 %) had a mRS at 4–5 and 4/26 (15.4 %) had a mRS 0. From 3 months till 1 year, functional outcome continued to improve as reported previously (Fig. 2) even for patients treated with invasive procedures such as craniectomy. Recently, the outcome and intracranial lesions of 69 patients with 45 treated by craniectomy were reported by Ferro et al. [6], with no information on ICU admission and strategy of care. The authors showed a good mRS 0–3 for 79 % with 16 % of them dying. The cohort of 44 craniectomy reported by Aaron et al. [13] had 59.3 % of mRS 0–3 at 3 months, a proportion that increased to 96.2 % after 1 year. Again, little information was given on ICU admission and treatment, which limits the comparison. Endovascular treatment was performed on 9 patients in our study for which 6 cases were considered successful. Our mortality rate for such patients was similar (33.3 %) to the one reported in 2008 using endovascular technique [14].

### Strategy of care

In the absence of published ICU treatment in severe CVT, our results are difficult to compare. If the use of a multimodal neurological monitoring might logically help for therapies, this cannot be demonstrated. We believe it may help to reduce the delay for management decisions, especially for craniectomy and/or endovascular treatment. Since SvjO_2_ cannot be recommended and intracranial pressure monitoring exposes to intracranial bleeding, the use of transcranial Doppler velocity, blood pressure, careful control of arterial PaCO_2_ and neurological examination are key parameters to alarm on brain hypoperfusion. As an example, the trend for a decrease in mean cerebral artery diastolic velocity motivates to perform brain imaging and to discuss the benefit of invasive treatment, such as craniectomy and/or endovascular treatment as illustrated in the case report shown in Additional file 4. If anticoagulation by heparin is the hallmark treatment for CVT [15], even when intracranial hematoma is present, it might be difficult to manage. First, sometimes efficient anticoagulation can be obtained after a long delay (>48 h), during which thrombosis may worsen. A shift toward another therapy might be necessary, such as low molecular weight heparin and/or endovascular thrombolysis. A long delay to reach therapeutic anticoagulation may precipitate to death or might indicate decompressive craniectomy, especially in the presence of intracranial hematoma. Second, bleeding complications may occur as we observed for 8 patients having intracranial bleeding and 5 bleeding at different peripheral levels. However, after dose adjustment or shift to another anticoagulant, the anticoagulation was never stopped. If the use of LMW heparin has been suggested [16, 17], standard heparin appears safer for intensivist, since it can be neutralized if necessary.

Incidence of seizures was high (43.9 %) during ICU stay even when the patients were treated before arrival (recurrence 88.9 %, 16/18). This context may facilitate aspiration pneumonia or ICP elevation. Control of seizure episodes frequently required drug associations.

Craniectomy became in the last 5 years a credible treatment to reduce intracranial pressure and secondary ischemic lesions after severe CVT, helping the venous blood to circulate. Instead of arterial ischemia, the low level of venous pressure may push the decision to perform the craniectomy for relatively moderate ICP elevation. Cortical veins and collapsed veins may then recirculate, reducing venous congestion and improving venous blood flow in collaterals. In addition, it favors anticoagulant to reach the thrombosed venous side [8, 18]. The positive outcome of craniectomy in this and previous reports [6, 19–21] supports its interest to treat severe CVT. Consequently, the positioning of this procedure on the therapeutic decision tree will move from the rescue indication to a more regular treatment for deterioration of venous congestion despite anticoagulation [7].

The interest of endovascular therapies to improve survival rate and functional outcome remains speculative [14, 22, 23]. If the recanalization of thrombosed profound vessels sounds a reasonable goal to obtain a good outcome [24–26] as observed in our study, it is not clearly demonstrated yet. The ongoing randomized clinical trial (TO-ACT) comparing thrombolysis (with or without thrombosuction) + heparin therapy *vs.* heparin alone will perhaps answer this question [27]. Decompressive craniectomy plus thrombolysis could be associated in the most severe cases, as we report in the case Additional file 4 [28, 29].

### Limits of the study

First, our study is a one-center study with limited number cases, even the largest published in ICU. This disadvantage can be balanced by the homogenous strategy of care for a Reference Center. The ICU team is then well trained to care severe brain injury patients including CVT and to initiate meeting after warning from clinic or multimodal monitoring items to discuss potential additional interventional therapy. Second, it is a retrospective analysis, which cannot be easily prospective considering the low incidence of this disease. Third, the positioning of craniectomy and/or endovascular therapies cannot be finalized because of the small numbers of cases.

## Conclusions

Severe CVT is rare among intensive care patients and differs largely from other severe brain injury admitted in ICU. An early and aggressive medical strategy is essential to prevent intracranial pressure elevation and improve venous drainage, which may require decompressive craniectomy or endovascular treatment. Despite a very severe initial clinical presentation, it is remarkable to observe an acceptable rate of death and frequent acceptable functional prognosis, which continues to improve after 1 year, as opposed to arterial brain ischemia.

## Additional files

**Additional file 1.** Risk factors for CVT in ICU from our cohort. Risk factors were classified according to conditions described in the literature. The association of tobacco and oral contraception was less frequent than in the literature. The 3 cases had solid cancer. Hematologic disorders were as follows: sickle-cell disease, myeloproliferative disorder, chronic lymphoid leukemia, thrombocytemia, polycythemia, multiple myeloma and autoimmune thrombotic purpura. Miscellaneous cases included diseases such as Behçet disease.

**Additional file 2.** Localization of venous thrombosis with mention for lateral sinus, comparing ICU survivors and deaths. p < 0.05 was considered as statistically significant.

**Additional file 3.** Comparison of admission characteristics and supportive therapies intensity between groups having craniectomy, endovascular treatment or not.

**Additional file 4.** Summary of therapeutic decisions for a severe CVT case based on multimodal monitoring and imaging. Figure 2a shows low transcranial Doppler diastolic velocities (<10 cm/s) with a low cerebral perfusion pressure (almost 40 mmHg; high ICP)), which did not improve after decompressive craniectomy. A collegially decided thrombectomy improved cerebral hemodynamic at day 3, allowing to stop sedation and to evaluate clinical symptoms. Figure 2b shows the initial venous-induced ischemia (hypo density), the subarachnoid hemorrhage with cerebral edema with narrowed ventricles (first picture). After decompressive craniectomy (second picture), ICP was persistently elevated in relation to hemorrhagic transformation and cerebral edema. Cerebral arteriography showed a non-recanalized superior sagittal sinus (SSS) despite adequate anticoagulation (third picture). After in situ thrombectomy and continuous thrombolysis, the flow in the SSS was re-established with a concomitant decrease in ICP (fourth picture).

## Abbreviations

aPTT: activated partial thrombin time
CSF: cerebral spinal fluid
CT: computerized tomography
CVT: cerebral vein thrombosis
GCS: Glasgow Coma Scale
ISCVT: International Study on Cerebral Vein and Sinus Thrombosis [3]
ICU: intensive care unit
ICP: intracranial pressure
IQ: interquartile
MRI: magnetic resonance imaging
mRS: modified Rankin score
MRV: magnetic resonance venography
SAPS II: Simplified Acute Physiology Score II
