# Erratum to: Outcome of a cohort of severe cerebral venous thrombosis in intensive care

**DOI:** 10.1186/s13613-016-0209-6

**Published:** 2016-11-02

**Authors:** Benjamin Soyer, Marco Rusca, Anne‑Claire Lukaszewicz, Isabelle Crassard, Jean‑Pierre Guichard, Damien Bresson, Joaquim Mateo, Didier Payen

**Affiliations:** 1 Department of Anesthesiology and Critical Care and SMUR, Hôpital Lariboisière, AP-HP, 2 Rue Ambroise Paré, 75010 Paris, France; 2 Université Diderot Sorbonne Paris Cité, Paris, France; 3 Département Hospitalo-Universitaire (DHU) Neuro-Vasculaire, P R E S Paris Sorbonne Cité, Paris, France; 4 Service of Neurology, Hôpital Lariboisière, AP-HP, Université Paris 7 Denis Diderot, Paris, France; 5 Department of Neurovascular Imaging, Hôpital Lariboisière, AP-HP, Université Paris 7 Denis Diderot, Paris, France; 6 Service of Neurosurgery, Hôpital Lariboisière, AP-HP, Université Paris 7 Denis Diderot, Paris, France

## Erratum to: Ann. Intensive Care (2016) 6:29 DOI 10.1186/s13613-016-0135-7

After publication of the original article [1], the author found an error in the fundamental result, the two parts involved and the corrections are as follows:

In the Result of the Abstract “After 12 months, 92 % of survivors (23/25) had a mRS between 0 and 3”. The correct sentence should be “After 12 months, 88.5 % of survivors (23/26) had a mRS between 0 and 3”.
