# Supplementary material for: Outcome of a cohort of severe cerebral venous thrombosis in intensive care
Source: Ann Intensive Care. 2016 Apr 12;6:29. doi: 10.1186/s13613-016-0135-7 (PMC4828343; doi:10.1186/s13613-016-0135-7)
Supplement: Supplementary file 1 — Additional file 1. Risk factors for CVT in ICU from our cohort. Risk factors were classified according to conditions described in the literature. The association of tobacco and oral contraception was less frequent than in the literature. The 3 cases had solid cancer. Hematologic disorders were as follows: sickle-cell disease, myeloproliferative disorder, chronic lymphoid leukemia, thrombocytemia, polycythemia, multiple myeloma and autoimmune thrombotic purpura. Miscellaneous cases included diseases such as Behçet disease. [file 13613_2016_135_MOESM1_ESM.pdf]

ADDITIONAL FILE 1

Outcome of a cohort of severe cerebral venous thrombosis in Intensive Care

Risk factors of CVT in ICU from our cohort. Risk factors were classified according to conditions described in literature. The association tobacco and oral contraception was less frequent than in literature. The 3 cases had solid cancer. Hematologic disorders were: sickle cell disease, myeloproliferative disorder, chronic lymphoid leukemia, thrombocytemia, polycythemia, multiple myeloma and auto immune thrombotic purpura. Miscellaneous cases included diseases such as Behçet disease.

Risk factors	All CVTs n=41 (%)	ICU Deaths n=10 (%)	ICU Survivors n=31 (%)	p value
Not identified	10 (24.4)	3 (30)	7 (22.6)	0.68
Oral contraception and hormonal treatment	9 (21.5)	3 (30)	6 (19.5)	0.66
Cancer related	7 (17.1)	1 (10)	6 (19.5)	0.65
Tumor	3 (7.3)	1 (10)	2 (6.5)	1.00
Radiotherapy	2 (4.9)	-	2 (6.5)	1.00
Chemotherapy	2 (4.9)	-	2 (6.5)	1.00
Hematologic disorders	9 (21.5)	1 (10)	8 (25.8)	0.41
Thrombo-embolic disease (personal and familial)	8 (19.5)	1 (10)	7 (22.6)	0.65
Miscellaneous	7 (17.1)	2 (20)	5 (16.1)	1.00
Genetic pro-thrombotic conditions	5 (12.2)	1 (10)	4 (12.9)	1.00
Protein S deficiency	2 (4.9)	1 (10)	1 (3.2)	0.43
MTHFR mutation	3 (7.3)	1 (10)	2 (6.5)	1.00
Pregnancy and post partum	5 (12.2)	2 (20)	3 (9.7)	0.58

Smokers	1 (2.4)	-	1 (3.2)	1.00
≥ 2 risk factors	9 (21.5)	2 (20)	7 (22.6)	1.00
