# Supplementary material for: Outcome of a cohort of severe cerebral venous thrombosis in intensive care
Source: Ann Intensive Care. 2016 Apr 12;6:29. doi: 10.1186/s13613-016-0135-7 (PMC4828343; doi:10.1186/s13613-016-0135-7)
Supplement: Supplementary file 2 — Additional file 2. Localization of venous thrombosis with mention for lateral sinus, comparing ICU survivors and deaths. p < 0.05 was considered as statistically significant. [file 13613_2016_135_MOESM2_ESM.pdf]

ADDITIONAL FILE 2

Outcome of a cohort of severe cerebral venous thrombosis in Intensive Care

Localization of venous thrombosis with mention for lateral sinus, comparing ICU survivors and deaths. $p<0.05$ was considered as statistically significant.

Topography of CVT	All CVTs n=41 (%)	ICU Deaths n=10 (%)	ICU survivors n=31 (%)	p value
Lateral Sinus	33 (80.5)	10 (100)	23 (74.2)	0.16
Left	17 (41.5)	6 (60)	11 (35.5)	0.27
Right	10 (24.4)	2 (20)	8 (25.8)	1.00
Both	6 (14.6)	2 (20)	4 (12.9)	0.62
Superior Sagittal Sinus	22 (53.7)	6 (60)	16 (51.6)	0.72
Cortical Veins	13 (31.7)	3 (30)	10 (32.3)	1.00
Straight Sinus	11 (26.8)	3 (30)	8 (25.8)	1.00
Deep Venous Network	11 (26.8)	2 (20)	9 (29.0)	1.00
Galien Vein	5 (12.2)	-	5 (16.1)	0.31
Deep Cerebral Vein	6 (14.6)	2 (20)	4 (12.9)	0.62
Jugular Vein	5 (12.2)	1 (10)	4 (12.9)	1.00
Others	12 (29.3)	3 (30)	9 (29.0)	1.00
Torcular	6 (14.6)	2 (20)	4 (12.9)	0.33
Sigmoid Sinus	5 (12.2)	1 (20)	4 (12.9)	1.00
Inferior Sagittal Sinus	1 (2.4)	-	1 (3.2)	0.56
