# Supplementary material for: Outcome of a cohort of severe cerebral venous thrombosis in intensive care
Source: Ann Intensive Care. 2016 Apr 12;6:29. doi: 10.1186/s13613-016-0135-7 (PMC4828343; doi:10.1186/s13613-016-0135-7)
Supplement: Supplementary file 3 — Additional file 3. Comparison of admission characteristics and supportive therapies intensity between groups having craniectomy, endovascular treatment or not. [file 13613_2016_135_MOESM3_ESM.pdf]

ADDITIONAL FILE 3

Outcome of a cohort of severe cerebral venous thrombosis in Intensive Care

Comparison of admission characteristics and supportive therapies intensity between groups having craniectomy, endovascular treatment or not. $p<0.05$ was considered as statistically significant. Data were expressed as median and 25th-75th interquartiles or as proportion. CVT: cerebral vein thrombosis. ICU: intensive care unit. SAPS II: simplified acute physiologic score II. GCS: Glasgow coma scale. aPTT: activated partial thromboplastin time. CSF: cerebral spinal fluid. IV: intravenous. MAP: mean arterial pressure. Min: minimal. mRS: modified Rankin score.

	Craniectomy n=16	No craniectomy n=25	p value	Endovascular treatment n=9	No endovascular treatment n=32	p value
Age (years)	47.5 (33.3-50.8)	47 (22-53)	0.66	47 (42-50)	47 (22.8-53)	0.66
Sex female (%)	14 (87.5)	16 (64)	0.15	7 (77.8)	22 (68.8)	0.70
SAPS II	41.5 (33.5-55.8)	40 (32-44)	0.37	42 (42-45)	36.5 (31.8-45.8)	0.12
Delay for diagnosis (days)	2.5 (1-5.5)	3 (1-8)	0.73	3 (2-6)	3 (1-7.3)	0.73
Delay for ICU admission (days)	1.5 (1-2.3)	1 (0-4)	0.41	2 (1-3)	1 (0-2.5)	0.42
GCS (lowest)	6.5 (5-8)	7 (5-9)	0.63	7 (6-7)	8 (5-9)	0.58
Seizure in ICU (n (%))	6 (37.5)	12 (48)	0.54	2 (22.2)	16 (50)	0.25
Mydriasis (n (%))	9 (56.3)	11 (44)	0.53	7 (77.8)	13 (40.6)	0.067
Hematoma (n (%))	15 (93.8)	17 (68)	0.066	8 (88.9)	24 (75)	0.65
Heparin before ICU (n (%))	12 (75)	13 (52)	0.19	7 (77.8)	18 (56.3)	0.44
aPTT ratio > 2 within 48h (n (%))	9 (56.3)	21 (84)	0.074	6 (66.7)	24 (75)	0.68
Craniotomy (n (%))	16 (100)	-	-	4 (44.4)	12 (37.5)	0.72
Endovascular treatment (n (%))	3 (18.8)	5 (20)	1.00	9 (100)	-	-
CSF shunt (n (%))	4 (25)	3 (12)	0.40	2 (22.2)	5 (15.6)	0.64
IV norepinephrine (n (%))	14 (87.5)	12 (48)	0.018	9 (100)	17 (53.1)	0.015
MAP min day 1 (mmHg)	78 (66-85)	74 (67-86)	0.86	86 (76-96)	74 (65-83)	0.16
MAP min day 2 (mmHg)	73 (70-81)	79 (71-85)	0.31	78 (73-91)	75 (70-83)	0.34
Mechanical ventilation (n (%))	16 (100)	21 (84)	0.14	9 (100)	30 (93.8)	1.00

Fluid balance day 1 (mL)	-195 (-528– +636)	-188 (-467– 28)	0.83	-292 (-430– -151)	-151 (-482– +625)	0.43
Fluid balance day 2 (mL)	609 (-725– +1263)	149 (-687– 672)	0.43	-113 (-723– +304)	401 (-667– +1075)	0.24
Natremia day 1 (mM)	138 (135-140)	138 (136-142)	0.47	139 (135-139)	138 (135-143)	0.77
Platelet count day 1 (10^9/L)	251 (198-321)	227 (181-286)	0.34	231 (213-243)	234 (179-307)	1.00
Glycemia day 1 (mM)	7.7 (6.5-8.4)	7 (5.6-7.9)	0.22	7 (6.3-7.7)	7.2 (5.9-8.4)	0.92
ICU deaths (n (%))	5 (31.3)	4 (16)	0.28	3 (33.3)	7 (21.9)	0.66
mRS at discharge	4 (3.8-6)	3 (2-4)	0.078	4 (3-6)	4 (3-5)	0.45
mRS at 3 months (n=23)	3 (3-4)	2 (1-3.3)	0.074	3.5 (2.3-4)	3 (2-4)	0.51
mRS at 12 months (n=18)	2 (2-3)	1.5 (1-3)	0.21	2.5 (1.3-3.8)	2 (1-3)	0.41
mRS at last follow-up	2 (1.8-6)	3 (0-6)	0.48	4 (1-6)	2 (1-6)	0.47
