# Supplementary material for: Outcome of a cohort of severe cerebral venous thrombosis in intensive care
Source: Ann Intensive Care. 2016 Apr 12;6:29. doi: 10.1186/s13613-016-0135-7 (PMC4828343; doi:10.1186/s13613-016-0135-7)

ADDITIONNAL FILE 4
Outcome of a cohort of severe cerebral venous thrombosis in Intensive Care

Summary of therapeutic decisions for a severe CVT case based on multimodal monitoring and imaging

Figure 2a shows low transcranial Doppler diastolic velocities (<10 cm/sec) with a low cerebral perfusion pressure (almost 40 mmHg; high ICP)), which did not improve after decompressive craniectomy. A collegially decided thrombectomy improved cerebral hemodynamic at day 3, allowing to stop sedation and to evaluate clinical symptoms.

Figure 2b shows the initial venous induced ischemia (hypo density), the subarachnoid hemorrhage with cerebral edema with narrowed ventricles (1st picture). After decompressive craniectomy (2nd picture) ICP was persistently elevated in relation with hemorrhagic transformation and cerebral edema. Cerebral arteriography showed a non-recanalized superior sagittal sinus (SSS) despite adequate anticoagulation (3rd picture). After in situ thrombectomy and continuous thrombolysis, the flow in the SSS was re-established with a concomitant decrease of ICP (4th picture).

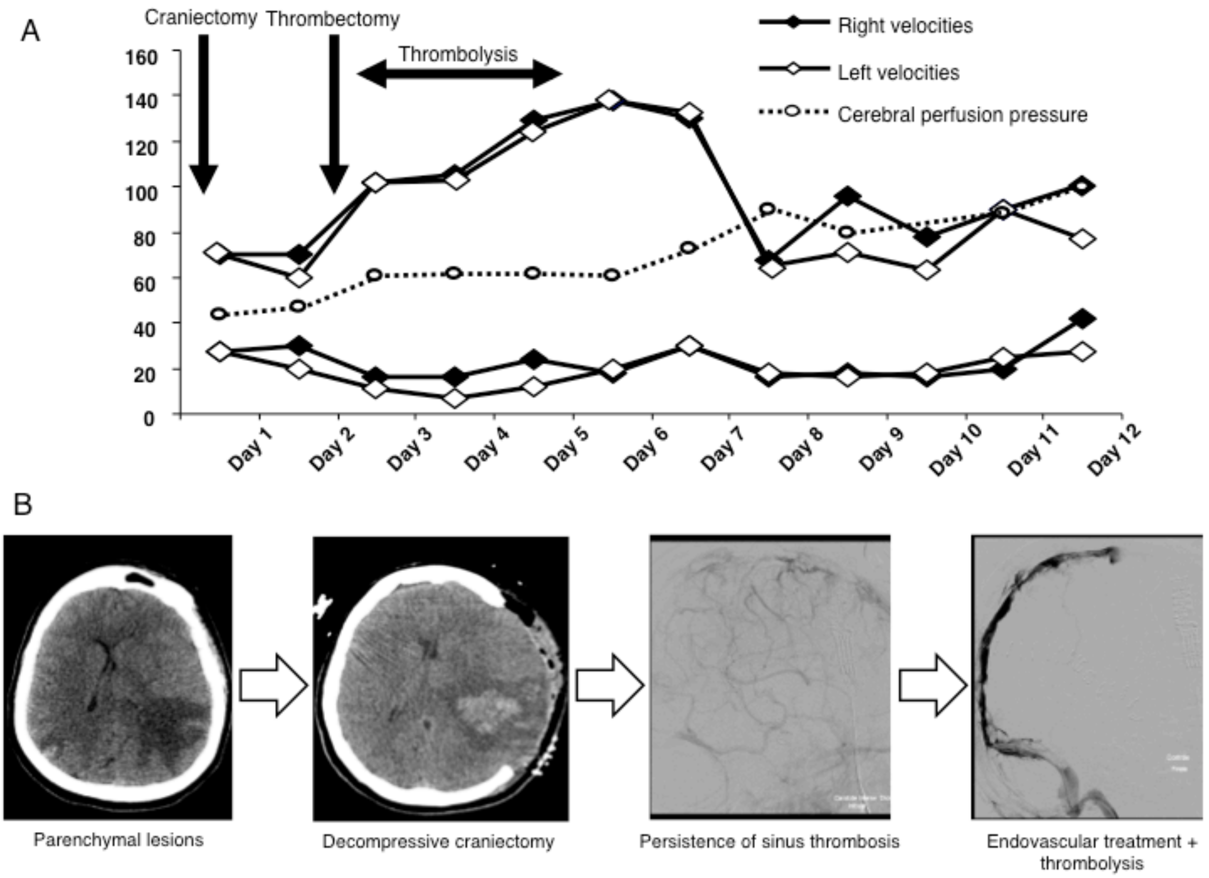

Supplement: Supplementary file 4 — Additional file 4. Summary of therapeutic decisions for a severe CVT case based on multimodal monitoring and imaging. Figure 2a shows low transcranial Doppler diastolic velocities (<10 cm/s) with a low cerebral perfusion pressure (almost 40 mmHg; high ICP)), which did not improve after decompressive craniectomy. A collegially decided thrombectomy improved cerebral hemodynamic at day 3, allowing to stop sedation and to evaluate clinical symptoms. Figure 2b shows the initial venous-induced ischemia (hypo density), the subarachnoid hemorrhage with cerebral edema with narrowed ventricles (first picture). After decompressive craniectomy (second picture), ICP was persistently elevated in relation to hemorrhagic transformation and cerebral edema. Cerebral arteriography showed a non-recanalized superior sagittal sinus (SSS) despite adequate anticoagulation (third picture). After in situ thrombectomy and continuous thrombolysis, the flow in the SSS was re-established with a concomitant decrease in ICP (fourth picture). [file 13613_2016_135_MOESM4_ESM.pdf]
